# Impact of early-life diet on long-term renal health

**DOI:** 10.1186/s40348-020-00109-1

**Published:** 2020-12-03

**Authors:** Eva Nüsken, Jenny Voggel, Gregor Fink, Jörg Dötsch, Kai-Dietrich Nüsken

**Affiliations:** Department of Pediatrics and Adolescent Medicine, Medical Faculty and University Hospital Cologne, University of Cologne, Kerpener Str. 62, 50937 Cologne, Germany

**Keywords:** Early-life diet, Maternal nutrition, Postnatal nutrition, Kidney development, Perinatal diet modification, Malnutrition, Renal programming, Kidney disease

## Abstract

In the last years, great advances have been made in the effort to understand how nutritional influences can affect long-term renal health. Evidence has accumulated that maternal nutrition before and during pregnancy and lactation as well as early postnatal nutrition is of special significance. In this review, we summarize epidemiologic and experimental data on the renal effects of perinatal exposure to energy restriction, low-protein diet, high-fat diet, high-fructose diet, and high- and low-salt diet as well as micronutrient deficiencies. Interestingly, different modifications during early-life diet may end up with similar sequelae for the offspring. On the other hand, molecular pathways can be influenced in opposite directions by different dietary interventions during early life. Importantly, postnatal nutrition significantly modifies the phenotype induced by maternal diet. Sequelae of altered macro- or micronutrient intakes include altered nephron count, blood pressure dysregulation, altered sodium handling, endothelial dysfunction, inflammation, mitochondrial dysfunction, and oxidative stress. In addition, renal prostaglandin metabolism as well as renal AMPK, mTOR, and PPAR signaling can be affected and the renin-angiotensin-aldosterone system may be dysregulated. Lately, the influence of early-life diet on gut microbiota leading to altered short chain fatty acid profiles has been discussed in the etiology of arterial hypertension. Against this background, the preventive and therapeutic potential of perinatal nutritional interventions regarding kidney disease is an emerging field of research. Especially individuals at risk (e.g., newborns from mothers who suffered from malnutrition during gestation) could disproportionately benefit from well-targeted dietary interventions.

## Introduction

**Fig. 1:**
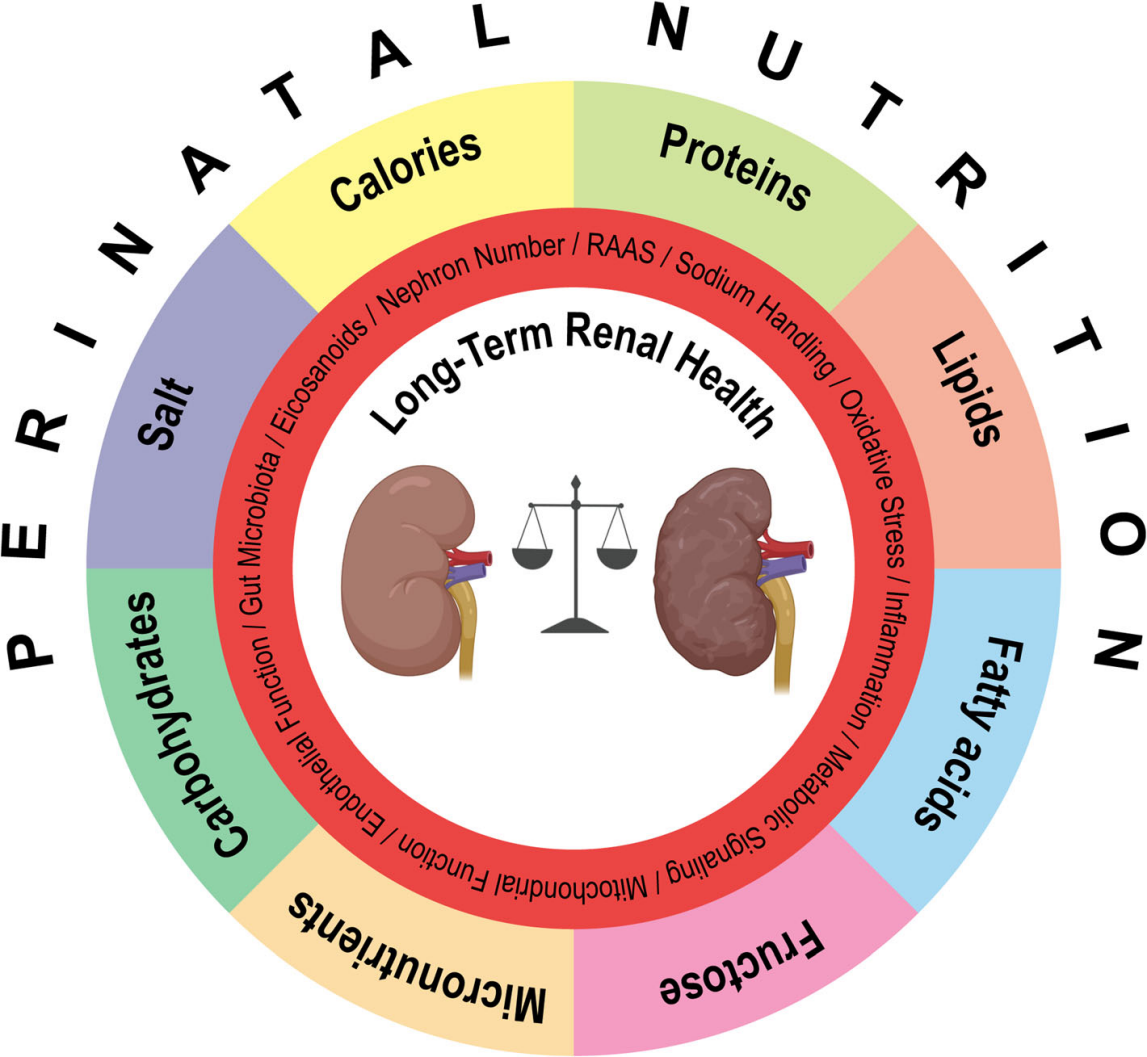
Schematic overview on the interplay between macro- and micronutrients and important developmental patterns/mechanisms involved in renal disease. RAAS, renin-angiotensin-aldosterone system. The figure was created using BioRender (www.biorender.com)

Animal studies on the influence of maternal nutrition on offspring kidney development can be found as early as in the 1960s [1]. First epidemiologic studies discussing the influence of early-life nutrition on risk of disease in adult life were published in the 1970s [2, 3]. Since then, great advances have been made in the effort to understand how nutritional influences during specific windows of development can affect long-term renal health. In this review, we summarize current knowledge of how energy intake and dietary composition of macronutrients and micronutrients during perinatal development act upon renal health (for an overview see Fig. 1).

## Energy restriction

Experimental studies on the effect of energy restriction are challenging to interpret since total energy restriction necessarily goes along with restriction of a varying amount and combination of macronutrients in most settings. In epidemiological studies, it is not even possible to define the exact composition of the diet. Despite these limitations, some interesting studies are hinting at possible effects of total energy restriction during early life on long-term renal health. Thus, individuals exposed to the Dutch Famine 1944/1945 during midgestation had an elevated risk of suffering from microalbuminuria during their fifth and sixth decade [4]. In Chinese women, exposure to the Chinese Famine 1959–1961 during fetal or early life was associated with an increased risk of proteinuria three decades later [5].

Rat offspring exposed to 50% maternal nutrient restriction throughout pregnancy compared to control ad libitum intake clinically developed reduced renal function, arterial hypertension, and increased proteinuria. Mechanistically, this was linked to reduced nephron count, endothelial dysfunction, oxidative stress, and RAAS dysregulation in adulthood [6, 7]. During kidney development, there was evidence for impaired ureteric bud branching and downregulation of proliferative pathways (ERK, p38 MAPK, Akt, PI3K, mTOR) in utero [8] and dysregulation of genes involved in PPAR signaling and glutathione metabolism during early life [9].

In sheep, periconceptional undernutrition, twin pregnancy, and maternal nutrient restriction to 50% of requirements during specific periods of kidney development were associated with altered gene expression of receptors regulating kidney growth in the offspring [10, 11]. Interestingly, animals exposed to 50% maternal nutrient restriction showed less pronounced renal inflammation when they were challenged by a high-fat diet postnatally [12].

In baboons, maternal nutrient restriction to 70% of controls during early to midgestation was associated with impaired tubule development, alterations in fetal renal mTOR signaling, and altered mitochondrial gene expression [13–15].

## Macronutrients

### Protein

Low-protein nutrition during gestation is probably the most widely used experimental setup to study the influence of early nutrition on adult renal health. Starting in the 1990s, it could first be shown in rats that low-protein diet throughout gestation results in reduced nephron count and arterial hypertension [16–19], pronounced deterioration of renal function with increasing age [20, 21], and increased susceptibility towards second hits [17, 22]. A multitude of molecular alterations mutually affecting each other has been identified in low-protein studies performed in rats in the meantime. Thus, quantitative and qualitative alterations of the renin-angiotensin-aldosterone system [18, 23–30], altered tubular salt handling [29, 31–35], and salt sensitivity of blood pressure [32, 36], as well as dysbalanced glucocorticoid metabolism [37], are important endocrine sequelae. Furthermore, there is evidence for enhanced inflammation [30] and oxidative stress [29, 30, 38] as well as altered prostaglandin metabolism [39] and endothelial [21] and mitochondrial dysfunction [40]. Dysregulation of AMPK, PPAR, and mTOR pathways may predispose towards obesity-associated kidney damage [41].

Importantly, it has been shown that postnatal nutrition significantly modifies the phenotype induced by maternal low-protein diet [42]. Thus, fostering low-protein pups to dams fed a control diet with normal protein content partially mitigates renal sequelae [43]. Conversely, healthy control pups raised in litters of 6–8 individuals by foster dams receiving low-protein diet developed arterial hypertension [44]. In another study, rat dams were protein-restricted during lactation only and litters were reduced to six males. Their offspring developed reduced nephron count, hyperfiltration, proteinuria, and altered salt handling associated with a dysregulation of angiotensin II signaling at 60 days of age [32]. In addition, both the phenotype and the molecular alterations are sex-dependent [37]. In some studies, it was shown that effects might even be transferred to subsequent generations [45].

Although most studies were performed in rats, similar results were published in mice [46, 47] and sheep [43, 48, 49]. Interestingly, maternal high-protein diet during gestation had no effect on renal morphology or function in the offspring [50].

### Lipids

Dietary lipids play a role in chronic kidney disease [51]. During kidney development, most studies have focused on the effect of excess dietary lipids. Conflicting results from these studies may rely on the finding that the fatty acid composition of dietary lipids has a major impact [52–54]. In addition, postnatal nutrition modifies the phenotype [55] and the dietary content of fructose should be taken into account since high-fat diet and Western-style diet have differential effects. Thus, studies influencing the effect of a “high-fat” diet are difficult to compare since the specific dietary interventions used to vary a lot.

In rats, maternal high-fat diet during gestation and lactation was associated with persisting upregulation of the renin-angiotensin system in adipose and renal tissue, increased oxidative stress markers, dysregulation of sodium transporters and circadian clock markers, and the development of arterial hypertension in adult life [56–58]. Perinatally, high-fat offspring presented with increased glomerular number which was no longer retraceable at 9 months of age [59]. Exposure to a modified high-fat diet rich in lipids containing saturated, mono-unsaturated, and n-6 polyunsaturated fatty acids in utero and until weaning resulted in vascular dysfunction, reduced renal Na+,K+-ATPase and reduced renin activity at 6 to 12 months of age. Renal stereology was not affected [60]. Exposure to both maternal and post-weaning high-fat diet (HF/HF) resulted in differentially composed gut microbiota and altered fetal concentrations of short chain fatty acids, which are known to affect blood pressure levels [61]. Treatment of HF/HF animals with the antioxidant resveratrol during young adult life prevented the development of arterial hypertension [62]. In another HF/HF study, tubular injury, impaired renal function, and increased expression of inflammatory markers were observed. These sequelae could be mitigated by n-3 fatty acid supplementation in the HF/HF group [54].

In mice, our group performed proteomic analyses of fetal kidneys shortly before birth. Proteins differentially expressed by maternal high-fat diet could be linked to eicosanoid metabolism, H2S-synthesis, transcription/translation, mitochondrial processes, and membrane remodeling [63]. In another mouse study, high-fat diet during gestation and lactation was associated with increased renal leptin signaling and decreased renal Akt/AMPK signaling at 3 weeks of age. Interestingly, at 10 weeks of age, leptin signaling was decreased in these animals [64]. Maternal high-fat diet restricted to the lactation period only had similar metabolic alterations in the offspring at 3 weeks of age but no effects at 10 weeks [64]. A study that combined maternal and post-weaning high-fat diet (HF/HF) resulted in albuminuria and increased renal triglyceride accumulation of the offspring going along with upregulation of markers indicative of inflammation, fibrosis, and oxidative stress. Experimentally induced overexpression of Sirtuin 1 partially mitigated these effects [65].

Remarkably, not only maternal but also paternal high-fat diet before mating can induce renal sequelae in the offspring. Thus, paternal high-fat diet in rats was associated with increased renal triglyceride accumulation and signs of tubular damage in adult male offspring, although in utero and postnatal conditions did not differ between groups [66]. Similar to sequelae seen in low-protein models, effects of high-fat diet in the offspring are sex-dependent [58].

### Fatty acids

In the Amsterdam Born Children and their Development (ABCD) study, low maternal serum concentrations of n-3 fatty acids (FA) and C20:3 n-6 (Dihomo-γ-linolenic acid, DGLA), and high maternal serum concentrations of trans fatty acids and C20:4 n-6 (arachidonic acid, ARA) were associated with an increased risk of giving birth to small for gestational age (SGA) infants [67]. In line with this, an Indian study reported a negative correlation of maternal ARA plasma concentrations and a positive correlation of maternal n-3 FA plasma concentrations with birth weight [68]. These findings are relevant for the kidney since epidemiological studies have shown that being small for gestational age is associated with an elevated risk of decreased renal function in young adulthood [69] and adverse course of glomerulopathies [70].

Postnatally, docosahexaenoic acid (DHA) concentrations in breast milk correlate with phospholipid FA composition of infant erythrocytes [71]. Dietary supplementation of the n-3 fatty acid DHA to the mother was shown to be an effective strategy to increase DHA breastmilk concentrations and increase omega-3 fatty acid availability during the neonatal period [71, 72]. In a mouse model, variation of dietary n-3/n-6 FA ratios during gestation and weaning was reflected in variation of kidney phospholipid FA composition [73]. Thus, perinatal availability of FA may have long-lasting consequences for the susceptibility towards kidney disease, since glycerophospholipid composition of organ membranes plays a role in a variety of pathologic conditions including cancer [74].

### Western-style diet

Perinatal and post-weaning Western-style diet (containing an increased amount of fat and fructose) in rats resulted in albuminuria, glomerulosclerosis, and tubulointerstitial fibrosis in adult life [55, 75] going along with an increased expression of inflammatory markers [75, 76].

### Fructose

High-fructose intake during gestation and lactation was associated with the development of arterial hypertension and increased expression of oxidative stress markers in rat offspring [77]. At 2 weeks of age, transcriptome analysis from renal rat tissue hinted at alterations of peroxisome proliferator-activated receptor (PPAR) signaling and glutathione metabolism [9]. Inhibition of soluble epoxide hydrolase in the offspring during the early postnatal period prevented the development of arterial hypertension. Mechanistically, this might rely on a regulatory effect of the arachidonic acid pathway leading to, e.g., an increase of vasodilatory epoxyeicosatrienoic acids (EETs) [77]. In another rat study, arterial hypertension induced by maternal high-fructose diet was attributed to dysregulation of gut microbiota as well as serum short chain fatty acids and their receptors in the offspring [78].

### Micronutrients

Human studies on the effect of micronutrients during early life were mainly performed in populations with a high percentage of suspected malnutrition. Thus, in a large randomized trial in Nepal, it could be shown that supplementation of the daily allowance of 15 minerals and vitamins in pregnant women was associated with a slightly lower blood pressure of their children at 2.5 years [79]. However, in a follow-up analysis studying Nepalese children at 6–8 years, no effect of maternal micronutrient supplementation on blood pressure levels could be demonstrated. Instead, there was evidence that supplementation of folic acid or a combination of folic acid, iron, and zinc during pregnancy reduced the prevalence of microalbuminuria in this age group [80]. In a similar study from Bangladesh, maternal micronutrient supplementation (daily allowance of 15 micronutrients minerals and vitamins) was even associated with a marginally higher diastolic blood pressure at 4.5 years of age [81].

Looking at single supplements, data is available for vitamin A, iron, and zinc. Thus, there was a positive correlation between maternal serum retinol concentrations and newborn kidney size at birth in a small cohort study from Egypt [82]. This would be in line with a study from rats, in which reduced vitamin A availability in utero induced low nephron count [83]. Similarly, iron restriction in rats caused a reduction of glomerular number in adult offspring [84]. In other studies, it could be shown that exposure to iron deficiency during gestation postponed nephrogenesis [85] and predisposed towards high-salt-induced arterial hypertension and mitochondrial dysfunction [86]. Deficiency during gestation was clinically associated with the development of arterial hypertension and decreased renal function of the offspring in experimental models. Histological and molecular analyses provided evidence for reduced nephron count and increased oxidative stress [87, 88].

### Salt

Studies on “high salt” and “low salt” diet during gestation and early postnatal development are highly variable regarding the exact amount of salt given.

In rats, both high- (3.0%) and low (0.07%)-salt diets during gestation and lactation were associated with arterial hypertension in adult male offspring at 5 months of age. Mechanistically, this was linked to low nephron count [89]. Similarly, a maternal diet containing 4% NaCl during gestation and lactation was associated with elevated blood pressure in male offspring in young adulthood. Interestingly, both male and female offspring were hypernatremic at this age despite being fed regular chow which was attributed to chronically increased corticosterone levels and altered gastrointestinal sodium handling [90]. A diet containing extremely high (8.0%) NaCl content during gestation (compared to 1.3% in controls) was shown to induce lower basal plasma renin activity, lower serum aldosterone, and reduced renal renin gene expression in male offspring at 12 weeks of age while blood pressure was elevated after high-salt challenge only [91]. Another study using the same salt exposure (8.0%) reported increased renal AT_1_:AT_2_-receptor and increased ACE:ACE2 expression ratios in the offspring [92]. In a study that supplemented salt not via food but via drinking water (1% NaCl) during pregnancy and lactation, male offspring showed alterations in the expression and activity of renal sodium transporters, increased infiltration with macrophages, increased deposition of collagen, and decreased AT_2_-receptor expression [93]. In the same setting (1% NaCl via drinking water), the development of arterial hypertension could be shown and next-generation RNA sequencing was performed to identify candidate genes for renal programming. In total, 272 genes were differentially expressed in high-salt offspring compared to controls, including genes belonging to clusters of cell adhesion molecules and complement and coagulation cascades [9].. Sheep offspring from mothers either exposed to a high-salt diet (14% compared to 2% NaCl) or fed saltbush (compared to dry pasture) during gestation had lower basal renin activity than their controls. In saltbush offspring, lower renin activity even persisted during post-weaning salt overload [94, 95].

### Molecular mechanisms

Interestingly, different modifications during early-life diet may end up with similar sequelae for the offspring. On the other hand, molecular pathways can be influenced in opposite directions by different dietary interventions during early life. In Table 1, we present an overview of early-life dietary modifications, known molecular effects on kidney development, and renal outcome.

**Table 1:** Overview of early-life dietary modifications and known molecular effects

Morphological/molecular effect	Early-life dietary modification
Reduced nephron count	Energy restriction [6, 7], low-protein diet [16, 17, 19], high- or low-salt diet [89], maternal deficiencies in vitamin A [83], iron [84], or zinc [87]
Dysregulation of the renin-angiotensin-aldosterone system (RAAS)	Energy restriction [7], low-protein diet [18, 23–27, 30], high-fat diet [56], and high-salt diet [91–93, 95]
Altered expression and/or activity of renal sodium transporters	Low-protein diet [29, 31–35], high-fat diet [58, 60], and high-salt diet [93]
Oxidative stress	Energy restriction [6, 7], low-protein diet [29, 30, 38], high-fat diet [56, 58, 62, 65], and high-fructose diet [77]
Inflammation	Low-protein diet [30], high-fat diet [54, 65, 75, 76], Western-style diet (high fat/high fructose) [75, 76], and high-salt diet [93]
Dysregulated metabolic signaling, (e.g., AMPK, mTOR, or PPAR signaling)	Energy restriction [8, 9, 13–15], low-protein diet [41], and high-fat diet [64]
Mitochondrial dysfunction	Energy restriction [13–15], low-protein diet [40], and high-fat diet [63]
Endothelial dysfunction	Energy restriction [6, 7], low-protein diet [21], and high-fructose diet [77]
Altered short chain fatty acid profile/dysregulated gut microbiota	High-fat diet [61] and high-fructose diet [78]
Prostaglandin metabolism	Low-protein diet [39]

### Secondary prevention and therapeutic potential of nutritional interventions

In recent years, there is growing interest in the therapeutic potential of nutritional interventions. Especially individuals at risk could disproportionately benefit from well-targeted dietary interventions. However, the number of dietary interventions to prevent renal disease is still limited.

In a human study from rural Nepal, supplementation of folic acid or a combination of folic acid, iron, and zinc during pregnancy reduced the risk of microalbuminuria in childhood [80]. Similarly, supplementation of selenium, folate, vitamin C, and vitamin E during 50% food restriction in rats prevented the development of arterial hypertension and endothelial dysfunction in the offspring. However, it did not protect against reduced glomerular number and impaired renal function in adulthood [6]. In rats exposed to low-protein diet during gestation, maternal supplementation of glycine prevented the development of arterial hypertension [96] and a single dose of vitamin A during midgestation prevented low nephron count [97]. However, in preterm but otherwise healthy baboons, early postnatal administration of vitamin A was not able to stimulate nephrogenesis [98]. Regulation of maternal gut microbiota by either probiotic or prebiotic strategies during maternal high-fructose diet prevented the development of arterial hypertension in the offspring. This effect was attributed to a regulation of gut microbiota as well as normalization of short chain fatty acids and their receptors in the offspring [70].

Taken together, these studies encourage further research on the preventive potential of dietary interventions. The better we learn to understand why an individual is at risk to develop kidney disease, the better we will be able to develop targeted nutritional interventions. So far, a couple of risk factors have been identified. In addition to adverse intrauterine conditions [99], ethnic background has been shown to influence renal function [100] and blood pressure [101] in childhood already. In part, this might be related to genetic factors. Thus, MYH9 polymorphisms [102] and APOL1 gene variants [103] have been identified as important factors contributing to the elevated risk of end stage renal disease in Americans of African ancestry compared to European ancestry. Interestingly, there is evidence from animal models that high-fat diet might upregulate MYH9 expression [104]. In a European population, variants at the UMOD locus were associated with advanced chronic kidney disease [105]. Thinking in terms of targeted nutritional interventions, this is relevant, since UMOD variants have also been linked to salt-sensitive hypertension [106]. Another aspect that needs to be considered in future studies on the preventive potential of dietary interventions is that optimal intake has not been defined for all nutrients for all age groups. For example, recent discussions about new regulatory standards on infant and follow-on formula for the European Union [107] demonstrate that there is still a need for studies defining optimal intakes of, e.g., n-3 and n-6 polyunsaturated fatty acids in infants.

## Conclusions

This review presents an overview of how maternal nutrition before and during pregnancy and lactation as well as early postnatal nutrition impact upon kidney development and long-term renal health. Adverse long-term effects have been documented for perinatal exposure to energy restriction, low-protein diet, high-fat diet, high-fructose diet, and high- and low-salt diet as well as micronutrient deficiencies. The exact renal phenotype significantly depends upon the timing of exposure. Important renal sequelae of altered macro- or micronutrient intakes include altered nephron count, blood pressure dysregulation, altered sodium handling, endothelial dysfunction, inflammation, mitochondrial dysfunction, and oxidative stress. In addition, renal prostaglandin metabolism as well as renal AMPK, mTOR, and PPAR signaling can be affected and the renin-angiotensin-aldosterone system may be dysregulated. Lately, the influence of early-life diet on gut microbiota leading to altered short chain fatty acid profiles has been discussed in the etiology of arterial hypertension. Importantly, it has been shown that postnatal nutrition significantly modifies the phenotype induced by maternal diet. Against this background, the preventive and therapeutic potential of perinatal nutritional interventions regarding kidney disease is an emerging field of research.

## Data Availability

Not applicable.

## Abbreviations

ACE: Angiotensin-converting enzyme
Akt: Protein kinase b
AMPK: Adenosine monophosphate-activated protein kinase
ARA: Arachidonic acid
AT_1_-receptor: Angiotensin 2 receptor type 1
AT_2_-receptor: Angiotensin 2 receptor type 2
DGLA: Dihomo-γ-linolenic acid
DHA: Docosahexaenoic acid
EETs: Epoxyeicosatrienoic acids
ERK: Extracellular signal-regulated kinases
FA: Fatty acid
H2S: Hydrogen sulfide
HF: High fat
mTOR: Mechanistic target of rapamycin
n-3 FA: Omega-3 fatty acid
n-6 FA: Omega-6 fatty acid
Na+,K+-ATPase: Sodium-potassium adenosine triphosphatase
NaCl: Sodium chloride
PI3K: Phosphoinositide-3-kinase
PPAR: Peroxisome proliferator-activated receptor
RNA: Ribonucleic acid
RAAS: Renin-angiotensin-aldosterone system
SGA: Small for gestational age
